# Dilatations des bronches associées à la broncho pneumopathie chronique obstructive: profil clinique et évolutif

**DOI:** 10.11604/pamj.2020.37.249.25023

**Published:** 2020-11-18

**Authors:** Chaima Habouria, Imen Bachouch, Nidhal Belloumi, Chahida Harizi, Fatma Chermiti, Soraya Fenniche

**Affiliations:** 1 Service de Pneumologie 4, Hôpital Abderrahmen Mami, Ariana, Faculté de Médecine de Tunis, Université Tunis, El Manar, Tunisie,; 2 Service d´Épidémiologie et de Statistique, Hôpital Abderrahmen Mami, Ariana, Faculté de Médecine de Tunis, Université Tunis, El Manar, Tunisie

**Keywords:** Broncho-pneumopathie chronique obstructive, dilatations des bronches, exacerbation, évolution, impact, Chronic obstructive pulmonary disease, bronchiectases, exacerbation, evolution, impact

## Abstract

**Introduction:**

l´association dilatations des bronches (DDB) et broncho-pneumopathie chronique obstructive (BPCO) a été proposée ces dernières années comme un nouveau phénotype potentiel de la BPCO en raison d´une présentation clinique et d´un pronostic différent. L'objectif de notre travail était d'étudier le profil clinique et les caractéristiques para cliniques des patients BPCO porteurs des DDB et de déterminer l'impact des DDB sur l'évolution de la maladie.

**Méthodes:**

étude rétrospective incluant 100 patients ayant une BPCO confirmée pris en charge entre 2014 et 2018 au service de pneumologie 4 à l´hôpital Abderrahmane Mami. Les patients ont été répartis en deux groupes: groupe 1: patients BPCO ayant des DDB (n=50) et groupe 2: patients BPCO sans DDB (n=50). Les deux groupes ont été appariés sur leurs caractéristiques épidémiologiques.

**Résultats:**

tous les patients étaient de sexe masculin, âgés en moyenne de 65,9 ans. Les patients BPCO et DDB avaient une fréquence plus élevée de cardiopathie ischémique (p=0,037), une dyspnée évaluée par le modified Medical Research Council plus élevée (mMRC≥2) (p=0,02), une obstruction bronchique plus sévère (p=0,005) et une fréquence plus élevée d´exacerbation aiguë (p<0,001) et d´hospitalisation (p=0,004). En étude multivariée, les facteurs indépendants associés à la présence de DDB étaient l´obstruction bronchique sévère (OR=9,16), le phénotype exacerbateur fréquent (≥2 exacerbations par an) (OR=1,91) et l'isolement de germes à l´examen cytobactériologique des crachats (OR=4,99).

**Conclusion:**

les patients BPCO et DDB pourraient ainsi représenter un phénotype distinct de la BPCO avec un pronostic plus réservé.

## Abstract (en)

**Introduction:**

in recent years, the combination of bronchiectases (BRs) and chronic obstructive pulmonary disease (COPD) has been described as a potential new phenotype of COPD due to its clinical features and different prognosis. The purpose of this study was to analyse the clinical profile and paraclinical features of COPD in patients with BRs and to determine the impact of BRs on disease progression.

**Methods:**

we conducted a retrospective study of 100 patients diagnosed with COPD and treated in the Department of Pneumology 4 at the Abderrahmane Mami Hospital between 2014 and 2018. Patients were divided into two groups: group 1: patients with COPD associated with BRs (n=50) and group 2: patients with COPD without BRs (n=50). Both groups were matched based on their epidemiological characteristics.

**Results:**

all patients were male, with an average age of 65,9 years. Patients with COPD associated with BRs had a higher rate of ischemic heart disease (p=0.037), more severe breathlessness assessed using the modified Medical Resaerch Council (mMRC≥2) (p=0.02), more severe bronchial obstruction (p=0.005) and a higher prevalence of acute exacerbations (p<0.001) and hospitalizations (p=0.004). In a multivariate study, independent factors associated with BRs were severe bronchial obstruction (OR=9.16), frequent exacerbator phenotype (≥2 exacerbations per year) (OR=1.91) and isolation of germs by cytobacteriological examination of sputum (OR=4.99).

**Conclusion:**

COPD associated with BRs could thus be a phenotype distinct from COPD and correlated with a more reserved prognosis.

## Introduction

La broncho-pneumopathie chronique obstructive (BPCO) est une maladie fréquente qui constitue un fardeau économique et social important. Elle représente une cause majeure de morbidité et de mortalité dans le monde [1]. Il s´agit d´une maladie hétérogène et complexe [2] dont la présentation clinique, la réponse au traitement et le pronostic varient considérablement d´un patient à un autre. Outre le degré d´obstruction bronchique et les exacerbations aiguës, d´autres facteurs ont démontré leur impact sur l´histoire naturelle de la BPCO. Parmi ces facteurs, les dilatations des bronches semblent avoir une valeur pronostique majeure chez les patients BPCO.

En 2014, les dilatations des bronches (DDB) ont été considérées pour la première fois comme une comorbidité de la broncho-pneumopathie chronique obstructive. La prévalence des DDB chez les patients BPCO est variable selon les différentes études allant de 4 à 72% [3]. Chez les patients ayant une BPCO modérée à sévère, la prévalence moyenne dépasse les 50% selon les séries [4]. Les DDB et la BPCO partagent de nombreuses caractéristiques communes aussi bien sur le plan physiopathologique, clinique que fonctionnel [5]. L´association des 2 pathologies pourrait définir un phénotype distinct de la BPCO [3,6]. Les particularités de ce sous-groupe de patients seraient des exacerbations plus fréquentes et plus sévères, une fonction respiratoire plus altérée mais aussi un risque plus important de colonisation bactérienne [7,8]. Cependant, l´association BPCO et DDB demeure peu étudiée dans notre contexte et l´impact des DDB sur l´histoire naturelle de la BPCO reste mal élucidé. L'objectif de ce travail est d´identifier les caractéristiques cliniques, radiologiques et fonctionnelles des patients ayant l´association BPCO et DDB et de déterminer l´impact des DDB sur l´évolution de la maladie.

## Méthodes

Il s´agit d´une étude rétrospective, comparative, incluant des patients suivis pour BPCO au service de pneumologie 4 à l´Hôpital Abderrahmane Mami en Tunisie, colligés sur une période allant de janvier 2014 à décembre 2018. Ont été inclus dans notre étude les patients ayant une BPCO au premier plan. Le diagnostic de la BPCO a été retenu sur les critères du GOLD associant une symptomatologie respiratoire chronique évocatrice et la présence d'un trouble ventilatoire obstructif (TVO) non complètement réversible à la spirométrie définie par un rapport volume expiratoire maximum à la première seconde (VEMS) / capacité vitale forcée (CVF) < 70% post bronchodilatation [1]. Seuls les patients ayant un suivi régulier d´au moins un an et ceux ayant bénéfice d´une tomodensitométrie thoracique (TDM) au cours de leurs suivi ont été retenus dans l´étude.

N´ont pas été retenus dans l´étude les patients ayant des DDB au premier plan anciennement connu et dominant le tableau et ceux ayant d´autres pathologies respiratoires associés (asthme, cancer broncho-pulmonaire, séquelles de tuberculose étendues, fibrose pulmonaire). Au total 100 patients ont été inclus et répartis en deux groupes: Groupe1 (G1) incluant 50 patients suivis pour BPCO au premier plan avec découverte de DDB au cours du suivi et Groupe 2 (G2) comportant également 50 patients suivis pour BPCO sans DDB. Les données ont été recueillies à partir des dossiers des patients. Nous avons noté pour chaque patient les caractéristiques sociodémographiques (l'âge et le sexe, tabagisme), les comorbidités, l´ancienneté de la BPCO et le délai de découverte des DDB dans le groupe1, le stade de la dyspnée d´effort selon l´échelle mMRC (modified Medical Research Council), le bilan fonctionnel respiratoire, la classification de la BPCO selon le GOLD, le traitement de fond de la BPCO, l´indication éventuelle d´une OLD ou VNI à domicile, les données de la TDM thoracique, le nombre d´exacerbation aiguë et d´hospitalisation par an, les caractéristiques cliniques et para cliniques des exacerbations aiguës ainsi que les traitements reçus, la durée du séjour, l´évolution.

Le diagnostic de DDB était confirmé dans tous les cas par les données d´une TDM thoracique consultable et montrant des bronches avec un ratio diamètre bronchique /diamètre artériel>1 ou une absence de diminution du calibre bronchique avec visualisation des bronches à moins d´ un cm de la plèvre [9]. L´étendue des DDB a été évaluée selon le nombre des lobes touchés. Ainsi, nous avons considéré comme DDB localisées, les dilatations des bronches qui touchent 1 ou 2 lobes du poumon, et par DDB diffuses un nombre de lobes atteints supérieur à deux. Une exacerbation aiguë (EA) de la BPCO a été définie selon le GOLD comme « Une aggravation aiguë des symptômes respiratoires nécessitant un traitement supplémentaire » [1]. Ont été considérés des exacerbateurs fréquents les patients ayant au moins deux épisodes d´exacerbations aiguës par an [1] Pour mieux étudier le profil clinique et para clinique des exacerbations aiguës, on a choisi de détailler dans chaque groupe une exacerbation aiguë sévère (par patient) ayant nécessité l´hospitalisation durant la dernière année de suivi. L´enquête bactériologique réalisée au cours de l´exacerbation a consisté en la réalisation d´un examen cytobactériologique des crachats (ECBC). Cet examen a été pratiqué à l´admission. Les critères de validité étaient la présence d´au moins 25 polynucléaires neutrophiles et de moins de 10 cellules épithéliales par champ. Le seuil de positivité retenu était de 10^7^UFC /ml [10].

Une colonisation bactérienne par *Pseudomonas aeruginosa* (PA) a été retenue devant la présence du PA sur au moins deux examens successifs à 3 mois d´intervalle dans les 12 derniers mois [11]. L´évaluation de l´état nutritionnel s´est basée sur le calcul de l´indice de masse corporelle (IMC) et les différents stades ont été définis conformément à la classification de l´OMS 2004 [12]. Devant l´indisponibilité du test de marche de 6 minutes pour tous les patients, on a choisi de calculé l´indice de BOD: Il s´agit d´un indice de BODE modifié où nous avons inclus trois paramètres: l´IMC, le VEMS (% de la valeur théorique), et le stade de la dyspnée évalué par l´échelle mMRC [13]. Les données ont été saisies et analysées à l´aide du logiciel SPSS (Statistical Package for the Social Sciences) version 25. Nous avons calculé des fréquences simples et des fréquences relatives (pourcentages) pour les variables qualitatives. Nous avons calculé des moyennes, des médianes et des écarts-types et déterminé l´étendue pour les variables quantitatives. La comparaison des moyennes sur les séries indépendantes a été effectuée au moyen du test de Student. La comparaison des proportions sur les séries indépendantes a été effectuée par le test du chi-deux (χ^2^) de Pearson, et en cas de non-validité de ce test par le test exact bilatéral de Fisher. Dans tous les tests statistiques, le seuil de signification a été fixé à 0,05. L´analyse multivariée a été réalisée par la régression logistique binaire. Elle a été effectuée pour les variables liées significativement à la présence des dilatations des bronches dont les « p » étaient <0,05. L´analyse multivariée a permis de calculer des Odds ratios ajustés, permettant d´identifier les facteurs liés de façon indépendante à la présence de DDB.

## Résultats

Au total, 100 patients présentant une BPCO ont été inclus et répartis en 2 groupes: groupe 1 (G1) comportant 50 patients ayant l´association BPCO et DDB et un groupe 2 (G2) incluant 50 patients suivis pour BPCO sans DDB. Les caractéristiques socio-démographiques des patients étaient comparables entre les deux groupes. Ils étaient tous de sexe masculin, vu le mode de recrutement dans le service et nous n´avons pas noté de différence significative concernant leurs moyennes d´âge (p=0,128). Les patients du G1 avaient un âge moyen de 67,5 ans (48-90 ans) versus 64,3 ans (42-84 ans) dans le G2. Le tabagisme était noté chez tous les patients. La consommation tabagique moyenne était comparable entre les deux groupes (62,3PA dans G1 vs 68,3PA dans le G2). Concernant les comorbidités, la cardiopathie ischémique était plus fréquemment retrouvée chez les patients du G1 (20% vs 6%, p=0,037). Nous n´avons pas noté de différence significative concernant la fréquence des autres comorbidités dans les deux groupes (Tableau 1). Concernant l´état nutritionnel, l´insuffisance pondérale (IMC< 18,5 Kg/m^2^) était plus fréquente chez les patients du G1 (32% vs 20%; p= 0,171). L´IMC moyen des patients du G1 était comparable à celui des patients du G2. Par ailleurs, la présence de DDB était associée à un indice de BOD plus élevé. En effet 48% des patients du G1 avaient un indice de BOD>4 versus 24% du G2 (p=0,006) alors que la majorité des patients du G2 avaient un indice de BOD <2 (38% du G2 versus 16% du G1; p=0,013) (Tableau 1).

**Tableau 1:** caractéristiques générales, cliniques spirométriques des deux groupes

Variables	Groupe 1: BPCO+DDB (n=50)	Groupe 2: BPCO sans DDB (n=50)	p
**Age** (ans)			
**Tabagisme** (PA)	67,5±10,5	64,3±10,03	0,128
**Comorbidités**, n (%)	62,3	68,3	0,341
HTA	8(16%)	9(18%)	0,79
Cardiopathie ischémique	10(20%)	3(6%)	0,037
troubles du rythme cardiaque	4(8%)	2(4%)	0,678
Diabète	6(12%)	8(16%)	0,564
Ulcère gastroduodénal	5(10%)	6(12%)	0,749
**Antécédents d´hospitalisation en réanimation**, n(%)	11(22%)	8(16%)	0,444
**Antécédent de recours à la ventilation mécanique**, n(%)	2(4%)	2(4%)	1
**IMC moyen (Kg/m^2^)**	21,08	22,7	0,062
**Insuffisance pondérale**, n(%)	16(32%)	10(20%)	0,171
**Indice de BOD**, n(%)			
Classe 1: 0-2	8(16%)	19(38%)	0,013
Classe 2: 3-4	18(36%)	19(38%)	0,76
Classe 3: 5-6	24(48%)	12(24%)	0,006
**Stade de la dyspnée mMRC**, n(%)			
mMrc1	4(8%)	12(24%)	0,029
mMrc2	25(50%)	26(52%)	0,841
mMRC3	20(40%)	9(18%)	0,015
mMRC4	1(2%)	3(6%)	0,307
mMRC≥2	46(92%)	38(76%)	0,02
**Grade GOLD**, n (%)			
Grade 2 (VEMS [50,80[	4(8%)	15(30%)	0,005
Grade 3 (VEMS [30,50[	24(48%)	16(32%)	0,102
Grade 4 (VEMS<30%)	22(44%)	19(38%)	0,542
Grades 3 et 4 (VEMS<50%)	46(92%)	35(70%)	0,005
**VEMS moyen (L)**	1,08±0,33	1,29±0,53	0,024
**Groupe ABCD**, n(%)			
Groupe A	(0%)	4(8%)	0,041
Groupe B	2(4%)	7(14%)	0,08
Groupe C	6(12%)	13(26%)	0,07
Groupe D	42(84%)	26(52%)	0,001
Groupes C et D	48(96%)	39(78%)	0,007

Abréviations: **PA**: paquet années. **n**: Nombre de patients. **HTA**: hypertension artérielle. **VEMS**: volume expiré à la première seconde lors d´une expiration forcée. **IMC**: indice de masse corporelle.

Les patients du G1 avaient une dyspnée d´effort plus intense à l´état stable: (mMRC≥2) chez 92% des patients du G1 versus 76% dans le G2 (p=0,02). Par ailleurs, 92% des patients du G1 avaient un VEMS<50% et étaient ainsi classés au stade III ou IV du GOLD versus 70% du G2 avec une différence statistiquement significative. Concernant le traitement de fond de la BPCO, les bronchodilatateurs de longue durée d´action étaient prescrits chez 46 patients (92%) du groupe 1 et chez tous les patients du groupe 2. La corticothérapie inhalée à dose modérée était prescrite chez 29 patients (58%) du G1 et 27 patients (54%) du G2. La présence de DDB était associée à une évolution significativement plus fréquente vers l´insuffisance respiratoire chronique avec le recours à l´OLD (p=0,039) (Tableau 2).

**Tableau 2:** caractéristiques radiologiques, thérapeutiques et évolution des deux groupes

Variables	Groupe 1 BPCO+DDB (n=50)	Groupe 2 BPCO sans DDB (n=50)	p
**Lésions de DDB**, n (%)	50 (100%)	0%	
**Epaississement des parois bronchiques**, n (%)	8 (16%)	6 (12%)	0,42
**Présence d´emphysème**, n (%)	35 (70%)	46(92%)	0,005
**Type de l´emphysème**, n (%)			
Emphysème centro lobulaire	31(88,6%)	38 (82,6%)	0,45
Emphysème panlobulaire	12(34,3%)	15(32,6%)	0,874
Emphysème paraseptal	24(68,5%)	30(65,2%)	0,755
Emphysème bulleux	6(17,1%)	19(41,3%)	0,02
Emphysème mixte	26(74,3%)	35(76,1%)	0,852
Emphysème diffus	24(68,6%)	26(56,5%)	0,269
**Traitement**, n(%)			
LABA+CSI	16(32%)	13(26%)	0,509
LAMA+CSI	5(10%)	2 (4%)	0,436
LABA+LAMA+CSI	4(8%)	12 (24%)	0,029
CSI+Théophylline	4 (8%)	0	0,056
Théophylline	29(58%)	20(40%)	0,072
**OLD**, n(%)	16(32%)	9(18%)	0,039
**VNI**, n(%)	9(18%)	5(10%)	0,249.
**Nombre de Décès**, n(%)	6(12%)	0	0,027

**Abréviations: n**: Nombre de patients. **LABA**: β2-mimétiques de longue durée d´action. **CSI:** corticostéroïdes inhalés. **LAMA:** anticholinergiques de longue durée d´action. **OLD:** oxygénothérapie de longue durée. **VNI:** Ventilation non invasive.

Sur le plan radiologique, dans le G1, les lésions de DDB étaient diffuses et bilatérales dans 60% des cas et localisées dans 40% des cas. L´aspect cylindrique était prédominant et était retrouvé dans 68% des cas. Dans les 2 groupes, des lésions d´emphysème étaient présentes dans respectivement 70% et 92% des cas (Tableau 2). Soixante-douze pour cent des patients du G1 étaient des exacerbateurs fréquents versus 38% dans le G2 (p=0,001). Les patients du G1 avaient également une fréquence d´exacerbations par an plus élevée (2,4 EA/an dans le G1 versus 1,48 EA/an dans le G2; p<0,001). De même, dans le G1, le nombre moyen d´hospitalisations pour exacerbations était plus élevé (p=0,004) (Tableau 3).

**Tableau 3:** caractéristiques cliniques biologiques et bactériologiques des exacerbations dans les 2 groupes

Variables	Groupe 1 BPCO+DDB (n=50)	Groupe 2: BPCO sans DDB (n=50)	P
**Exacerbateurs fréquents**, n (%)	36(72%)	19(38%)	0,001
**Moyenne d´EA /an**	2,4	1,48	<0,001
**Moyenne d´hospitalisation pour EA /an**	1,64	1,10	0,004
**Isolement du PA au cours du suivi**, n(%)	18(36%)	3(6%)	<0,001
**Colonisation par PA**, n (%)	7(14%)	0	0,012
**Profil gazométrique de l´EA**, n (%)			
IRA	37(77,1%)	22(56,4%)	0,04
IRA avec acidose respiratoire décompensée	14(29,2%)	7(17,9%)	0,224
IRA hypercapnique	9(18,8%)	7(17,9%)	0,924
**Profil biologique de l´EA**, n(%)			
Hyperleucocytose (GB>10000/mm^3^)	23(47,9%)	22(56,4%)	0,43
CRP>15mg /L	26(54,2%)	18(46,2%)	0,45
**Profil bactériologique de l´EA**, n(%)			
ECBC positif	28/46(60,9%)	6/29(20,7%)	0,001
*Pseudomonas aeruginosa*	15 /46(32,6%)	2 /29(6,9%)	0,01
**Recours à la VNI**, n(%)	14(29,2%)	9(23,1%)	0,522
**Transfert en réanimation**, n(%)	3(6,25%)	2(5,12%)	0,8
**Durée moyenne de l´hospitalisation** (Jours)	12,44	12,31	0,9

**Abréviations: n**: nombre de patients. **EA**: exacerbation aiguë. **PA**: *Pseudomonas aeruginosa*. **IRA**: insuffisance respiratoire aiguë.**CRP**: protéine C reactive. **ECBC**: examen cytobactériologiques des crachats.**VNI**: ventilation non invasive

Au cours de la dernière année de suivi, 48 exacerbations du groupe 1 et 39 exacerbations du groupe 2 nécessitant l´hospitalisation ont été inclus. Un tableau d´insuffisance respiratoire aiguë était noté chez 37 patients (77,1%) du G1 versus 22 patients du G2 (56,4%) (p=0,04) et l´étiologie bactérienne a été documentée chez 60,3% des patients du G1 versus 20% dans le G2 (p=0,001). Le germe le plus incriminé chez les patients du G1 était le PA, isolé dans 32,6% versus 6,9% dans le G2 (p=0,01). Une évolution vers la colonisation au PA était observée chez 7 patients du G1 (p=0,012) (Tableau 3). En étude multivariée, la présence d´un Trouble ventilatoire obstructif sévère à très sévère (VEMS<50%) (OR=9,16), l´isolement de germes à l´ECBC (OR=4,99) et le phénotype exacerbateur fréquent (OR=1,91) étaient les facteurs indépendants les plus associés à la présence de DDB chez les patients BPCO (Tableau 4).

**Tableau 4:** facteurs associés au groupe des patients BPCO et DDB dans le modèle d**´**analyse multivariée

Facteurs de risque	p en étude univariée	p en étude multivariée	OR	IC 95%
**Fréquence d´exacerbation ≥2**	0,001	0,269	1,91	[0,60-6,09]
**VEMS <50%**	0,024	0,016	9,16	[1,51-55,55]
**ECBC positif**	0,001	0,008	4,99	[1,51-16,50]

**Abréviations: VEMS**: volume expiré à la première seconde lors d**´**une expiration forcé. **ECBC**: examen cytobactériologiques des crachats

## Discussion

L´association BPCO et DDB semble très fréquente mais demeure sous diagnostiquée et peu étudiée. Notre étude avait pour objectif de dresser le profil clinique, radiologique et fonctionnel des patients ayant une BPCO et des DDB et de déterminer l´impact des DDB sur le cours évolutif de la maladie. Selon les résultats de notre étude, la présence de DDB Chez les patients BPCO semble avoir une influence péjorative sur l´histoire clinique de la BPCO. En effet, les patients ayant l´association BPCO et DDB avaient une fréquence significativement élevée de cardiopathie ischémique, des scores de dyspnée mMRC plus élevés, une fonction pulmonaire plus altérée, un risque plus important d´exacerbation et d´hospitalisation. En outre, les DDB augmentent significativement le risque d´isolement de germes à l´examen cytobactériologique des crachats, en particulier le PA.

La BPCO et les DDB sont deux affections du sujet âgé [14]. Dans une revue de la littérature, Martinez-Garcia a constaté que la moyenne d´âge des patients ayant l´association BPCO et DDB variait entre 62 ans et 73 ans [15]. En outre, dans la majorité des études, il ressort que les patients ayant l´association BPCO et DDB étaient plus âgés que les patients ayant seulement une BPCO [3,8,16,17]. Dans notre étude, les sujets porteurs de DDB (G1) avaient un âge légèrement plus avancé que ceux ayant seulement une BPCO. La moyenne d´âge était respectivement de 67,5 ans et de 64,3 ans, sans différence statistiquement significative. Concernant le sexe, vu le mode de recrutement dans le service, les patients inclus dans notre étude étaient tous de sexe masculin. Cela ne nous a pas permis de conclure quant à l´impact du sexe sur ce profil de patient. Néanmoins, dans la majorité des études, nous avons constaté que l´association BPCO et DDB était plus fréquente chez les sujets de sexe masculin [8,18-20]. Ce constat pourrait être expliqué par une intoxication tabagique plus importante chez la population masculine [8,15,19]. Le tabac principal facteur incriminé dans la BPCO pourrait aussi avoir un rôle clé dans le développement des dilatations des bronches chez les patients BPCO. En effet, en altérant les mécanismes de défense pulmonaire, il facilite l´installation des infections et favorise la persistance des germes dans les voies respiratoires, ce qui déclenche une cascade d´évènements menant à l´altération progressive de la paroi bronchique et l´apparition des DDB [3]. Dans notre étude le tabagisme était noté chez tous les patients. Contrairement aux données de la littérature [8,21,22], dans notre série, la présence de DDB n´était pas associée à un degré d´intoxication tabagique plus important.

La BPCO et les DDB considérées longtemps comme des maladies principalement respiratoires, font partie actuellement des maladies systémiques [23,24]. En effet, l´inflammation systémique constitue un facteur pathogène commun à ces deux états pathologiques et semble jouer un rôle déterminant dans le déclenchement et l´aggravation de certaines comorbidités comme les maladies cardiovasculaires, les comorbidités métaboliques, et aussi la cachexie [7]. Les données de la littérature concernant l´étude des comorbidités chez ce profil de patient sont limitées. Dans notre étude, la comparaison des 2 groupes n´a pas montré de différence significative concernant la présence de comorbidités, sauf pour les cardiopathies ischémiques qui étaient significativement plus fréquentes chez les patients porteurs de DDB (20% vs 6%; p=0,037). Nos résultats rejoignent ceux d´une étude comparant 15802 BPCO à 3955 patients ayant l´association DDB et BPCO, les auteurs ont constaté une prévalence significativement plus élevée d´insuffisance cardiaque chez les patients porteurs de DDB (p<0,001) [25]. Selon les auteurs, un tel constat pourrait s´expliquer par une majoration de l´inflammation systémique qui joue un rôle déterminant dans les maladies cardiovasculaires [25]. Selon les données de la littérature, la dénutrition touche 25 à 40% des patients BPCO et serait plus fréquente au cours des stades avancés de la maladie [26]. Par ailleurs, il ressort de plusieurs études que la présence de DDB chez les patients BPCO retentit négativement sur leur état nutritionnel [17-19]. Dans notre étude, l´IMC moyen était comparable dans les deux groupes. Toutefois, l´insuffisance pondérale était plus fréquemment retrouvée chez les patients porteurs de DDB (32% vs 20%; p =0,171).

Les patients BPCO porteurs de DDB sont plus symptomatiques à l´état stable. En effet le stade de dyspnée mMRC s´accroit en présence des dilatations des bronches [16,18,26,27]. D´ailleurs, c´est le cas de notre étude. En effet, un stade de dyspnée mMRC≥2 était plus fréquemment rapporté par les patients ayant l´association BPCO et DDB (92% versus 76%; p=0,02). Par ailleurs, dans la littérature [28,29], la présence de DDB chez les patients BPCO est associée à une fonction pulmonaire altérée qui pourrait s´expliquer en partie par une susceptibilité accrue aux exacerbations qui sont considérées responsables d´un déclin rapide de la fonction respiratoire [30]. Martínez-García MA a constaté dans une étude menée auprès de 92 patients BPCO que la prévalence des DDB dépasse 70% chez les patients ayant un VEMS <50% et qu´un TVO sévère à très sévère (VEMS<50%) est un facteur de risque indépendant qui multiplie de 3,87 le risque de développer des DDB [31]. Ces résultats sont concordants avec les nôtres. Dans notre étude, le VEMS moyen était significativement plus bas chez les patients porteurs de DDB (p=0,024). Nous avons également constaté une relation statistiquement significative entre la sévérité du TVO et la présence de DDB (p=0,005). En étude multivariée, un VEMS<50% était un facteur indépendant des autres facteurs, le plus associé à la présence de DDB (OR=9,16). En concordance avec nos résultats, dans la littérature, les DDB associées à la BPCO sont souvent de type cylindriques, bilatérales, prédominant au niveau des bases avec des scores de sévérité modérés [7,32,33]. L´aspect cylindrique est constaté dans 80 à 97% des cas selon les séries [26,31,34,35]. La prédilection de l´atteinte des lobes inférieurs s´expliquerait par l´effet de la gravité sur la stase et la rétention des sécrétions infectées [36]. L´approche radiologique pour évaluer l´étendue et la sévérité des DDB était variable selon les études. En effet, plusieurs études se sont basées sur le nombre de segments [31], ou des lobes touchés [34], d´autres ont utilisé des scores radiologiques tels que le score de Smith [5,19], et celui Bhalla modifié [22,37]. Quel que soit le score utilisé, les DDB étaient en fait généralement diffuses et bilatérales de sévérité modérée. Dans notre étude, en considérant le nombre de lobes touchés, nous avons constaté que les DDB étaient bilatérales et diffuses dans 60% des cas et localisées dans 40% des cas. L´imagerie chez les patients du G1, a montré également qu´en dehors des lésions de DDB, des lésions d´emphysème et d´épaississement de la paroi bronchique étaient constatées dans respectivement 70% pour les lésions d´emphysème et 16% pour l´épaississement de la paroi bronchique. Certains auteurs suggèrent que les DDB pourraient avoir un impact sur l'évolution de la BPCO au-delà des effets de l'emphysème et de l'épaississement de la paroi bronchique en altérant la clairance mucociliaire, entrainant ainsi la stase des secrétions et du mucus et favorisant par conséquent la colonisation bactérienne [38].

Il importe de souligner qu´il existe une hétérogénéité considérable dans les caractéristiques, la fréquence et l'évolution dans le temps des exacerbations chez les patients BPCO. Cette hétérogénéité ne peut pas être expliquée uniquement par le degré d'obstruction bronchique ou la sévérité du grade de la maladie. Wedzicha et Hurst ont démontré que la présence de DDB peut moduler et affecter la fréquence et la gravité des exacerbations [39]. Ainsi, dans la littérature il ressort que la présence de DDB chez les patients BPCO est associée à des exacerbations aiguës plus fréquentes, plus graves et plus prolongées [3,8]. Dans sa méta-analyse, incluant 14 études comparant des patients BPCO et DDB à des patients ayant seulement une BPCO, Du et al ont démontré que la présence de DDB multiplie de 1,9^7^ fois le risque d´exacerbations chez les patients BPCO (OR=1,97) [7]. Par ailleurs, dans une étude Thaïlandaise, les auteurs ont trouvé que la présence de DDB était associée de façon indépendante et significative au profil exacerbateur fréquent (OR=4,99) [20]. En outre, une autre étude a rapporté que la présence de DDB augmente de 3 fois le risque d´hospitalisation pour exacerbation aiguë (OR=3,07) [31]. Ces résultats concordent avec les nôtres puisque dans notre étude la présence de DDB était associée de façon significative aussi bien au phénotype exacerbateur fréquent (72% vs 38% avec p=0,001), à une fréquence d´exacerbation par an plus élevée (2,4 vs 1,48; p<0,001), qu´à une moyenne d´hospitalisation pour exacerbation par an plus élevée (1,64 vs 1,10; p=0,004). De plus, en étude multivariée, la présence de DDB représente un facteur indépendant associé au phénotype exacerbateur fréquent (OR=1,91). Selon les estimations actuelles, un quart des exacerbations de BPCO est d´origine bactérienne, et un quart d´origine virale. Cependant la cause infectieuse n´est pas toujours facile à prouver chez les patients BPCO. Dans la littérature, la présence de DDB chez les patients BPCO est associée à un risque significativement plus élevé d´isolement de germes au cours des exacerbations aiguës [21,31,16]. Dans notre étude, l´étiologie bactérienne a été documentée chez 60,9% des patients BPCO et DDB ayant eu un ECBC contre 20,7% des patients BPCO sans DDB (p=0,001). En plus, dans notre série, en étude multivariée, la présence de DDB a été retenue comme facteur indépendant associé à un risque plus élevé d´isolement de germes à l´ECBC (OR=4,99). Par ailleurs, le germe le plus incriminé au cours de l´exacerbation chez nos patients BPCO et DDB était le PA. D´autres études appuient ce constat. Dans la méta-analyse de Du, comme dans celle de Ni, il a été démontré que la présence de DDB chez les patients BPCO augmente significativement le risque d´isolement des microorganismes potentiellement pathogènes dans les expectorations des patients ayant l´association BPCO et DDB (OR respectivement de 3,76 et de 7,33) et particulièrement le PA avec un OR respectivement de 4,5 et de 3,5 [7,8].

Concernant l´évolution de la BPCO, dans notre étude, l´évolution vers l´insuffisance respiratoire chronique nécessitant le recours à l´OLD était significativement plus fréquente chez les patients ayant l´association BPCO et DDB. Cette évolution, témoignant de l´impact négatif des DDB sur l´évolution de la BPCO, est rapportée par plusieurs autres études [16,31,34]. Ces résultats sont néanmoins à interpréter compte tenu de certaines limites. Le caractère mono centrique et rétrospectif constitue les limites majeures de notre étude. Malgré ces limites, à travers ce travail nous avons pu démontrer l´impact négatif des DDB sur l´histoire naturelle de la BPCO dans notre contexte. A notre connaissance, les données tunisiennes sont peu nombreuses à ce sujet. Pourtant, l´association des deux pathologies est fréquente selon la littérature. Il importe de signaler par ailleurs, qu´il existe peu de données concernant le traitement de fond des patients ayant l´association BPCO et DDB. De plus, à l´heure actuelle, il n´y a pas de recommandations ni de consensus pour la gestion de ce groupe de malades. Les bronchodilatateurs, qu´ils soient β-adrénergiques ou anticholinergiques, ne semblent pas poser de problème en raison de leur grande efficacité et sécurité chez les patients atteints de BPCO [15]. Toutefois, la corticothérapie inhalée (CSI) du fait de son effet immunosuppresseur doit être évitée chez les patients ayant l´association BPCO et DDB. En effet, plusieurs études ont constaté que l´utilisation des CSI chez les patients BPCO était associée à une augmentation de la charge bactérienne dans les voies respiratoires [40] avec un risque accru de pneumonie [41]. En outre, le risque de pneumonie chez les patients BPCO sous CSI était plus élevé en présence de DDB [42]. Par ailleurs, les macrolides utilisés pour leurs propriétés anti-inflammatoires étaient largement étudiés aussi bien chez les patients DDB que chez les BPCO. Néanmoins, leurs indications, leurs bénéfices thérapeutiques ne sont pas encore déterminés chez les patients ayant l´association BPCO et DDB.

## Conclusion

Selon les résultats de notre étude, nous avons constaté que la présence de DDB chez les patients BPCO constitue un facteur de mauvais pronostic. En effet, la présence de DDB chez les patients BPCO augmente le risque des exacerbations aiguës, d´isolement de germes à l´ECBC et aggrave la fonction respiratoire. Ainsi, nos résultats rejoignent ceux de la littérature et appuient l´existence de ce phénotype particulier. L´impact négatif des DDB sur l´évolution naturelle de la BPCO justifie l´intérêt majeur de l´identification précoce de cette comorbidité. Nous insistons sur l´importance de la réalisation de la TDM thoracique au cours du suivi des malades BPCO, et nous la recommandons chez les exacerbateurs fréquents aux stades avancés de la maladie. D´autre part, la pratique d´un ECBC de façon périodique permet de détecter à temps et de traiter les infections à PA. De même un dépistage des comorbidités pouvant aggraver le pronostic notamment les comorbidités cardiovasculaires et la dénutrition est fortement recommandé vue leur fréquence plus élevée chez ce groupe de patients. D'autres études sont nécessaires pour une meilleure appréciation de l´efficacité des traitements disponibles pour ce sous-groupe spécifique de patients notamment l´utilisation des corticostéroïdes inhalés chez les patients BPCO porteurs de DDB, de même la prescription des macrolides au long cours chez ces patients en particulier.

### Etat des connaissances sur le sujet

- L´association BPCO et DDB pourrait définir un nouveau phénotype de la BPCO;
- Les DDB pourraient avoir une influence sur l´histoire clinique et évolutive des patients BPCO.

### Contribution de notre étude à la connaissance

- A notre connaissance, cette étude constitue l´une des premières études réalisées dans une population africaine qui fournit des données utiles concernant l´association BPCO et DDB;
- La présence de DDB chez les patients BPCO est responsable d´une aggravation de la fonction respiratoire, d´un risque plus important d´exacerbations bactériennes et d´hospitalisations;
- Les patients BPCO et DDB pourraient ainsi représenter un phénotype distinct de la BPCO avec un pronostic plus réservé.
